# A Miniaturized Therapeutic Chromophore for Multiple Metal Pollutant Sensing, Pathological Metal Diagnosis and Logical Computing

**DOI:** 10.1038/srep27115

**Published:** 2016-06-07

**Authors:** Bhimsen Rout

**Affiliations:** 1 Organic Chemistry Division, Institute of Chemical and Engineering Sciences, A*STAR, 138665-Singapore

## Abstract

The efficacy of a miniaturized unimolecular analytic system is illustrated. The easily accessible therapeutic chromophore “temoporfin”, which responds differentially to bound metals at multiple wavelengths of Q-band absorption using chemometric analysis, expeditiously detects and discriminates a wide range of metals regarded as priority pollutants in water and hence may also be used for diagnosis of medically relevant metals in human urine. The molecule was further investigated as an electronic logic device, e.g. keypad lock device, to authorize multiple highly secure chemical passwords for information protection.

The designing of analytical systems to sense multianalytes at the molecular level^1^,^2^,^3^,^4^,^5^ and exploiting them as computational devices to mimic logic gates and circuits^6^,^7^,^8^,^9^,^10^,^11^,^12^,^13^ has attracted special attention in the last decades. Ever since the invention of the “lab on a molecule” technology using two metal ions and proton, based on the “receptor-per-analyte” paradigm by A. P. de Silva^6^, many diagnostic devices were developed to detect multiple metals with high sensitivity^14^,^15^,^16^,^17^. Subsequently, using cross-reactive sensor arrays and multivariate data analysis, multianalyte detection was done with molecular ‘noses/tongues’ to detect metal ions^18^,^19^,^20^, proteins^21^,^22^,^23^ and other analytes straightforwardly with much advance detection capabilities^18^,^19^,^20^,^21^,^22^,^24^,^25^,^26^,^27^,^28^. However, the lack of physical integration limits its simplicity, speed and probably hinders application as complex logic gate devices.

To date, miniaturization to a single-molecule sensor has been achieved using, e.g., multichannel sensing to detect different metal ions^29^,^30^,^31^,^32^,^33^, shape shifting molecules to detect saccharides^34^,^35^ or chromophoric sensors^36^,^37^,^38^ to detect metal ions^37^,^38^. Their potential was demonstrated by the detection of multianalytes in solution. However, each of these methods has its own intrinsic limitations such as the use of different instruments or light sources during measurements, single binding site preferences for most analytes, restricted to produce signals from UV to lower visible region (200–500 nm) and incubation times. This impedes a standardized approach of analyte determination, thus encumbering distinct applicability, e.g. molecular computation.

Recently, development of multiple dye and receptor-integrated sensors has received widespread interest. For example, a combinatorial unimolecular sensor as an advanced “lab on a molecule” was synthesized by covalently attaching four fluorophores to a common cis-amine-L-proline platform for detection of counterfeit medications^4^; this sensor was also evaluated as keypad lock device^39^,^40^,^41^,^42^,^43^,^44^,^45^,^46^,^47^ to generate multiple chemical passwords^39^. Likewise, Eric T. Kool and coworkers developed a very elegant concept of tetramer-length oligodeoxy-fluoroside (ODF) sensor sequences attached to microbeads, each of which contains four different dye units for the detection of various metal ions^48^,^49^,^50^. These examples^1^,^2^,^3^,^4^,^5^,^6^,^7^,^8^,^9^,^10^,^11^,^12^,^13^,^14^,^15^,^16^,^17^,^18^,^19^,^20^,^21^,^22^,^23^,^24^,^25^,^26^,^27^,^28^,^29^,^30^,^31^,^32^,^33^,^34^,^35^,^36^,^37^,^38^,^39^,^40^,^41^,^42^,^43^,^44^,^45^,^46^,^47^,^48^,^49^,^50^ demonstrate that modern approaches aimed at high analytical capacity and easy applicability have markedly advanced multi-analyte detection and molecular computation. Still, there is scope for an alternative sensor to both concepts of (1) a multi-dye integrated sensor that involve tedious synthetic assembling or (2) a single-molecule driven multichannel sensor, based on diverse measuring parameters.

Here we describe a miniaturized analytical system consisting of a single universal “receptor-cum-reporter”, temoporfin (**1**), which is an easily accessible therapeutic chromophore and which responds differentially to bound metal ions at multiple wavelengths of Q-band absorption across the far visible to near IR region (500–700 nm). Employing chemometric analysis to the differential responses, expeditious detection and discrimination of metals and mixtures of metals, e.g. occurring as occupational pollutants in solutions, has been demonstrated. The potential of temoporfin was further investigated as a 2-digit electronic keypad lock device to create highly secure multiple chemical passwords for information protection. Thus, the capability of the new diagnostic system takes miniaturization of analytical device to the next level in the field of both sensing and computing.

Metal ions such as chromium, manganese, mercury, cadmium, zinc, copper, silver, lead, and nickel are known to cause adverse health effects and are regarded as ‘priority pollutants’ listed by the United States Environmental Protection Agency (USEPA)^51^,^52^. Several fluorophoric sensors based on porphyrin were developed for few specific metal ion detection^53^,^54^,^55^,^56^,^57^, logic gate device construction^58^,^59^,^60^, protein recognition^23^ and nanopore formation^61^,^62^ mostly using soret band (B-band) for excitation. A very limited number of chromophoric sensors^32^,^63^,^64^,^65^,^66^,^67^,^68^ was reported for the detection of few metal ions^63^,^64^,^65^,^66^ using single porphyrin derivatives. However, these porphyrin-based sensors have not been explored with respect to all the Q-band absorption peaks and the wide range of applications to sense a variety of metals, different concentrations and mixtures of metals in solution, and applications in complex logic devices.

## Result and Discussion

The spectral characteristics of the metalloporphyrin is governed primarily by the size and position of metal ions with respect to the cavity of the ligand (as anticipated by Barnes and Dorough)^69^ and secondly, with the electronic structure of the metal centers (as proposed by Gouterman)^70^,^71^. The spectral difference is further contributed by steric effects with substituents on α, β, meso position of the porphyrin ring^72^, aggregation^73^,^74^,^75^,^76^ and axial ligation^77^.

**Figure 1:**
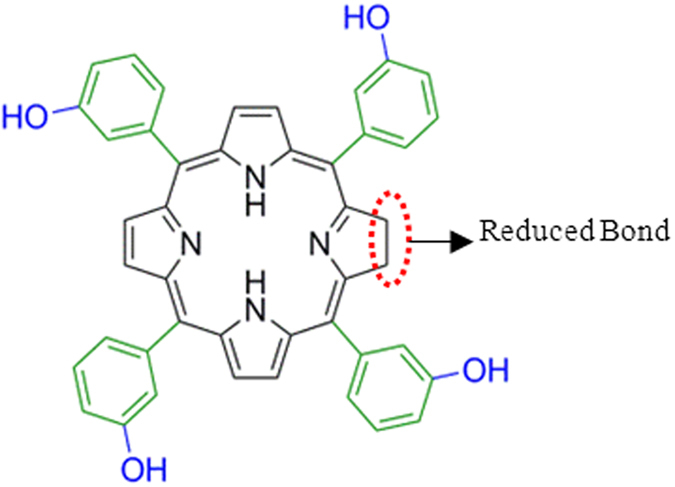
Structure of the chromophoric sensor 1, temoporfin [5,10,15,20-tetrakis(3-hydroxyphenyl) chlorin].

To be able to identify and discriminate different metal ions, we have chosen a less symmetric and commercially available chlorin molecule, namely temoporfin [5,10,15,20-tetrakis(3-hydroxyphenyl)chlorin] **1** (Fig. 1), a clinically approved photodynamic therapeutic drug, to generate substantial changes at four intense Q-band absorption peaks upon metallation. The conventional higher symmetric metalloporphyrin system generates only two Q-band peaks (see supporting information, Fig. S1). The selection of meta-hydroxyphenyl substitution at the meso position of **1** can induce sufficient steric hindrance to generate more conformational perturbation by different analytes. In addition, the hydroxyl group on the phenyl ring can coordinate to neighboring central metal ions axially to induce additional deformation by M-OH bonding. Altogether, the steric effects of meso substitution, the influence of size and charge of metal ions on binding cooperativity, the extent of axial M-OH bonding and aggregation in the analytical media increase the conformational dynamics of metallochlorin. This structural change results in unsymmetrical vibrations along the X- and Y-axis of sensor which can be utilize for the generation of distinct analyte-specific Q-band spectral signatures.

**Figure 2:**
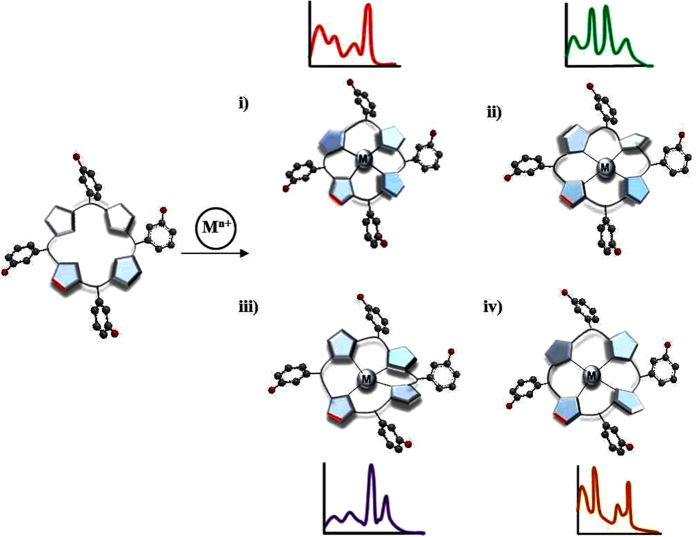
Illustration of function of sensor 1. Selected examples out of many known possible out-of plane distorted metallochlorin complexes (top view) are pictorially represented that can create unique absorption signature: (**i**) dome, (**ii**) wave, (**iii**) saddle and (**iv**) ruffle. The red line on the chlorin backbone implies a reduced bond; M^n+^ = metal ions.

Furthermore, metalloporphyrins are known to exist in the most frequently observed out-of-plane conformations such as dome, saddle, ruffle and wave (see supporting information, Fig. S2 and Fig. 2). For example, dome conformation (complex **i**) was observed when metal ions of larger size such as mercury or lead were complexed with porphyrin as these metal ions are not accommodated in the core of the porphyrin but sit just above the ring, thus making all four nitrogen of the pyrrole moieties face the metal ion [for other deformations detail; see supporting information, Fig. S2]. In addition to these, other less frequently occurring conformations such as propeller, helical and their combinations (e.g. sadruf, rufwav) including few in-plane distorted conformations was also observed^78^,^79^,^80^. Analogous circumstances with higher asymmetry and deformation would arise in the case of molecule **1** owing to reduced bond on the chlorin backbone and steric hindrance of the meso substitution. Figure 2 illustrates substituted chlorin molecule upon complexation with metals having different sizes, charges, binding constants resulted distortion at four pyrrole moieties of the ring in different ways such as dome, saddle, ruffle, wave etc., that brings different molecular vibrations along X- and Y-axis of the molecule and hence, generate different Q-band absorption spectra. The possibility of existence in a single unsymmetrical distorted conformation (e.g. **i**–**iv**) and/or a combination of several deformations (e.g. **i,** and/or **ii**, and/or **iii**, and/or **iv**; common for a mixture of metals) will determine the variation of resultant Q-band absorption patterns.

The sensor **1** has four intense Q-band peaks which appear to be due to unsymmetrical molecular vibration along the X- and Y-axis. To get the best response from all the Q-band absorption wavelengths, different solvent conditions were screened; Dulbecco’s PBS buffer/methanol (1/9), pH = 7.3, was found as a preferential solvent (see supporting information, Fig. S3). Using only soret band (B-band) of **1**, many metal ions produced identical spectra except Cu^2+^ and Fe^3+^ (see supporting information, Fig. S4).

**Figure 3:**
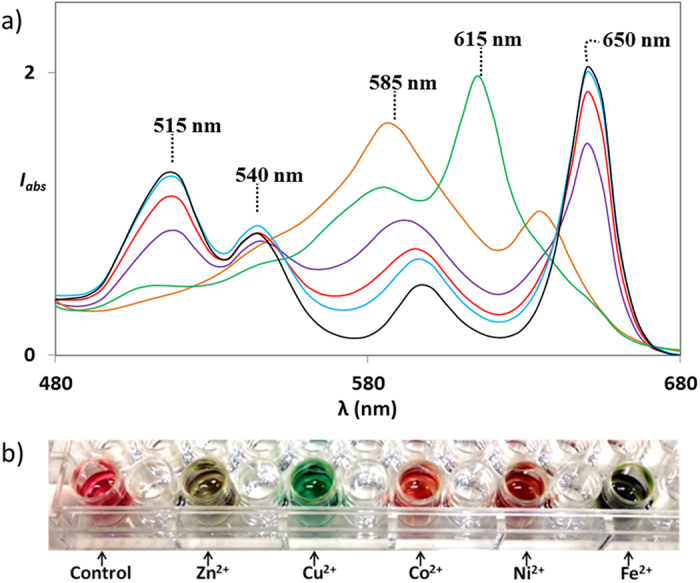
(**a**) Representative Q-band absorption signatures generated by **1** (black) upon the addition of 2 mM of Zn^2+^ (purple), Cu^2+^ (green), Mn^2+^ (red), Hg^2+^ (blue), and Fe^3+^ (orange). (**b**) Image of plate reader indicates visual colour change of **1** (control, pink) with different metals [Zn^2+^ (olive green), Cu^2+^ (green), Co^2+^ (red), Ni^2+^ (brown), and Fe^3+^ (dark olive green)].

The efficiency of sensor **1** was demonstrated by incorporating different metals that generated unique optical signatures at multiple Q-band absorption wavelengths (Fig. 3a). Readily distinguishable absorption peaks at 585 nm for Fe^3+^ (orange) or 615 nm for Cu^2+^ (green) were observed after exposing sensor **1** to these metal ions. These unique spectral features made Fe^3+^ and Cu^2+^ distinguishable from the other metal ions tested in this study. Another characteristic pattern of reduced absorption intensities at 515 and 650 nm along with an increase at 585 nm was noted for Zn^2+^ (purple). For Hg^2+^ (blue), similar read-outs as the sensor alone were observed, but with a distinctly increased intensity at around 585 nm. The typical absorption signature of Mn^2+^ (dark red) is perceived with a reduced intensity at 515 nm and an increased intensity at 585 nm. These results indicate that each metal ion has its unique Q-band absorption pattern in the far visible to near IR region (Fig. 3a).

**Figure 4:**
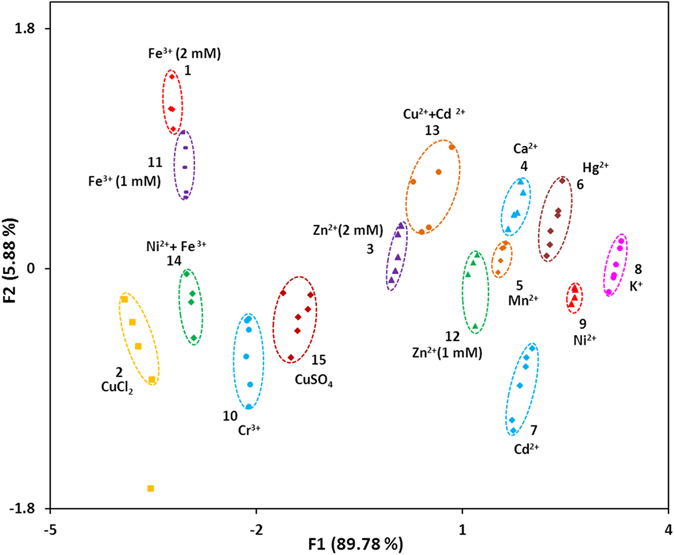
PCA plot of Q-band absorption patterns generated by sensor **1** upon the addition of metal ions (2 mM, **1–10**), half conc. of metal ions (1 mM, **11–12**), mixture of metal ions (1 mM each, **13–14**) and diff. anion bearing metal (2 mM, **15**).

Conspicuous colour change observed by the addition of selected metal ions further supports visual discrimination ability of sensor **1** (Fig. 3b), as has been reported for a few other sensors^48^,^49^,^50^,^53^,^81^,^82^. However, solely colour-based detection has its limitation with regard to the detection of unknown samples of identical colour-generating ‘different metal species’, ‘different concentration of a particular metal’, ‘different counter anion of a metal’, as well as ‘mixture of metals’ including non-colour generating metals. To discriminate between a range of metals and different concentrations and mixtures of metals, the absorption spectra were recorded several times for each analyte. The intensities were analysed at six different wavelengths (515 nm, 545 nm, 595 nm, 600 nm, 615 nm and 650 nm) employing principal component analysis (PCA, Fig. 4). The PCA was able to detect and differentiate between ten different metal ions (**1**–**10**) and different concentrations of metal ions (**1** vs. **11**, **3** vs. **12**), different counter anion bearing metal ions (**2** vs. **15**) as well as mixture of metal ions (**2** and **7** vs. **13**, **9** and **11** vs. **14**) with 95.7% discrimination ability (Fig. 4). An unknown training set of samples was analysed with 90% accuracy (see supporting information, Table S1).

**Figure 5:**
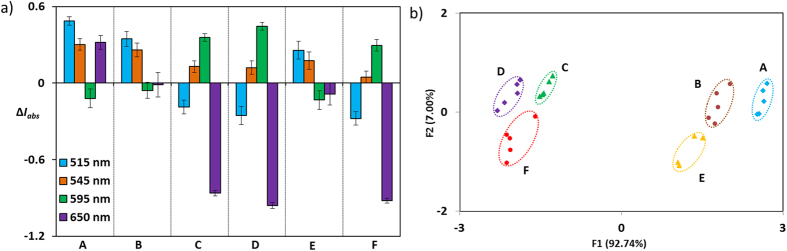
(**a**) Change in Q-band absorption intensities (Δ*Iabs* = 1 − *Iabs*) of **1** at four different wavelengths upon addition of urine containing A) Chromium (0.53 mg/mL); B) Chromium (0.26 mg/mL); C) Cobalt (0.48 mg/mL); D) Cobalt (0.24 mg/mL); E) mixture of Chromium (0.53 mg/mL) and Cobalt (0.48 mg/mL); F) mixture of Chromium (0.27 mg/mL) and Cobalt (0.24 mg/mL); error bars represent standard deviations. (**b**) Corresponding PCA plot.

Apart from the identification of metals, the ability of our analytical system was demonstrated by the detection of various pathologically important metal ions^83^,^84^ and its mixtures. For example, for the simultaneous detection of chromium and cobalt metal ions in human urine during post-surgery recovery of metal-on-metal hip resurfacing of young patients^83^,^84^, there is a need of simpler and high-throughput diagnostic methods than the commonly used complicated method of detection^85^,^86^,^87^,^88^. Urine is such a biological continuum that has a strong background noise below 450 nm. Beyond signalling at far visible to near IR region (500–700 nm), molecule **1** has an advantage to capture metal ions from the non-specific interaction of urine proteins to provide much accurate results. Introducing human urine loaded with chromium and cobalt metals at different concentration and different ratios to **1**, the changes in Q-band intensity at four different wavelengths (515 nm, 545 nm, 595 nm, 650 nm) were determined for six urine-metal samples (A–E) (Fig. 5a). The intensities at these wavelengths were analysed with PCA which successfully discriminates six different spiked samples (Fig. 5b). The unknown samples were identified with 93% success rate (supporting information, Table S2). Admittedly, the clinical samples are hundred fold less than metal concentration used in our prototype study; however, this can be overcome by collecting urine sample for several hours and subsequently reducing the volume of urine by lyophilisation. As our prototype operates in absorption mode (simple and quick), the detection limit of our sensor is 100 uM to 1 mM and can be further minimized by using different derivatives of temoporfin that has more aqueous solubility.

Interestingly, during the detection of mixture of metals (see supporting information, Table S1), it was observed that some absorption measurements of metal-mixtures were inconsistent probably due to the slow kinetics of these metal complexations and to the short incubation period (5 min) after analyte addition. Therefore, the observed slow kinetics was further exploited by adding right combination of metals in sequence and by extending incubation period (10 min, 5 min after each addition). A few metals were tested using different combination sequences (see supporting information, Fig. S7): Two different absorption spectra were reproducibly obtained with a different order of addition of two metals (M0 = Fe^3+^ and M1 = Cu^2+^) to the molecule **1**. This opened the perspective to investigate the electronic 2-digit keypad lock device^39^,^40^,^41^,^42^,^43^,^44^,^45^,^46^,^47^ nature of molecule **1**.

After the invention of a first molecular keypad lock by Margulies *et al.*^40^, many molecular keypad locks^41^,^42^,^43^,^44^,^45^,^46^,^47^ were reported that create only single default chemical passwords. Recently a much advanced multi-dye integrated combinatorial keypad lock^39^ was reported with advanced computing capability to distinguish and discriminate multiple chemical passwords using principal component analysis (PCA). Although, PCA may not be an optimal method to perform an operation that a 2-input priority AND gate can execute and also computationally demanding which needs higher computer capacity in comparison to two physically button wired digital lock, it has the excellent ability to distinguish multiple passwords generated by one molecular lock. Temoporfin **1** offers a miniaturized unimolecular keypad lock device that does not involve multiple dyes avoids complicated chemical synthesis and utilizes only metal ions (M0 = Fe^3+^ and M1 = Cu^2+^) as two input keys in four different ways (M0M0, M1M1, M0M1, M1M0); in this way four distinguishable absorption patterns are obtained.

**Figure 6:**
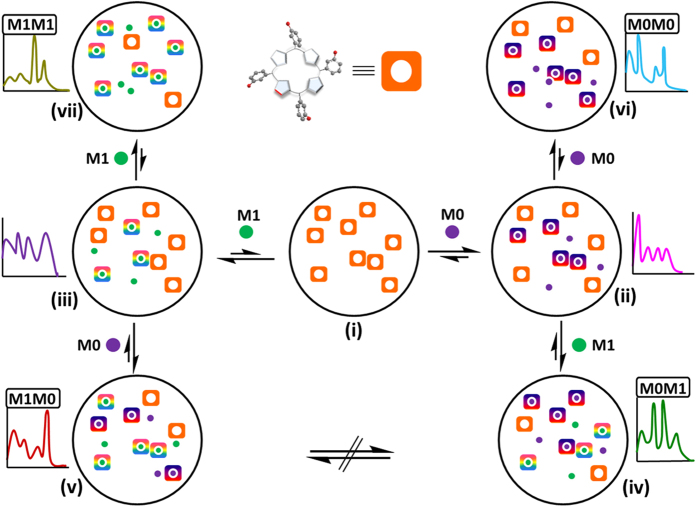
Illustration of the possible intermediate states that can be formed upon addition of two metals (green circle, M0 and purple circle, M1) in different order (M0M1 vs. M1M0) and in different concentrations (M0 vs. M0M0 and M1 vs. M1M1). The orange square represents molecule **1**.

**Figure 7:**
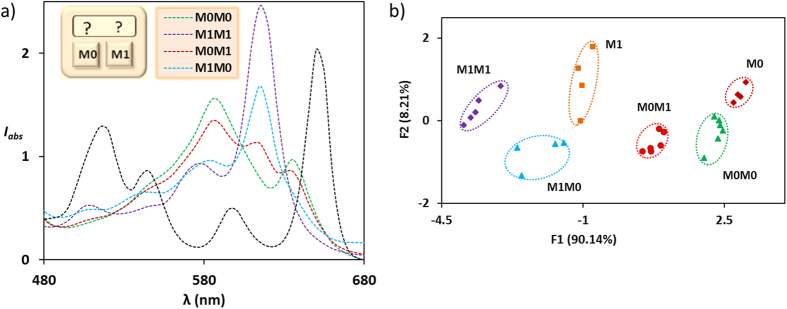
(**a**) Q-band absorption pattern generated by **1** (black) upon addition of two input keys Fe^3+^ (M0) and Cu^2+^ (M1) in four different 2-digit entries M0M0 (green), M1M1 (purple), M0M1 (red), M1M0 (blue). (**b**) PCA of all six 1-digit and 2-digit chemical passwords.

With metals, kinetic intermediates can be expected due to their different charges, sizes, ability to bind receptors in distinct stoichiometries (e.g. 1:1, 1:2), in different binding conformations (e.g. dome, wave, planar), all of which increase the possibility of entrapment in various local energy minima. Figure 6 illustrates how the strong binding of the first metal (i.e., M0 or M1) to receptor **1** (i.e., complexes **ii** and **iii**) followed by a weaker binding of the second metal can result in a kinetically stable complex (i.e., complexes **iv** and **v**) whose conversion to thermodynamic stable product takes a prolonged reaction time. Figure 6 represents how an increase of the metal concentration (i.e. complex **ii** and **vi** or **iii** and **vii**) results in different absorption spectra, as the ratio between bound (more green circle or purple circle attached orange squares) and unbound temoporfin (orange squares) is increased. This was demonstrated by addition of each metal (M0 or M1), followed by a second addition of the same input signal generates different absorption pattern for M0M0 or M1M1 code entries (Fig. 7a). However, changing the second input to different metal type alters the type of complexes formed (i.e., complex **ii** and **iv** or **iii** and **v**) as well as ratio between them, and hence generates resultant distinct absorption signature for M0M1 or M1M0 code entries (Fig. 7a). The conversion to thermodynamic stable product (i.e. a constant ratio between green circle attached orange squares, purple circle attached orange squares, and unbound orange squares) takes prolong reaction time.

The PCA of the absorption intensities at six different wavelengths (510 nm, 550 nm, 575 nm, 590 nm, 615 nm and 640 nm) shows that the unimolecular chromophoric sensor **1** can discriminate among all six possible permutations of 1- and 2-code entries, namely, M0, M1, M0M0, M1M1, M0M1, M1M0, akin to an equivalent 2-digit electronic keypad device (Fig. 7b). Unlike a multi-dye integrated combinatorial lock^39^, these chemical passwords were created by the unimolecular miniaturized device **1** without assembling any individual logic (dye) component. These chemical passwords are doubly protected and unbreakable as they adopt both the principle of electronic digital locking and biometric pattern locking.

## Conclusion

The differential response and correlative signals at several Q-band absorption wavelengths of therapeutic chromophore **1** equals the condition as if many signalling dyes were arrayed within our unimolecular sensor and contributed to a correct discrimination analysis for distinguishing different metals and authorizing multiple chemical passwords expeditiously. The results presented show how effective a unimolecular receptor/sensor system can be when used in combination with chemometric tools. Hence, therapeutic chromophore **1** competes efficiently with multiple-dye integrated sensors or devices, multichannel sensors and array based sensors, and takes analytical device miniaturization^6^,^7^,^8^,^9^,^10^,^11^,^12^,^13^,^14^,^15^,^16^,^17^,^18^,^19^,^20^,^21^,^22^,^23^,^24^,^25^,^26^,^27^,^28^,^29^,^30^,^31^,^32^,^33^,^34^,^35^,^36^,^37^,^38^,^48^,^49^,^50^,^51^,^52^,^53^,^54^,^55^,^56^,^57^,^58^,^59^,^60^ a step further with easy accessibility and diversification using an already existing concept of pattern generation and chemometric analysis. The multi-metal receptive nature of the unimolecular therapeutic device **1** further increases its potential for the construction of a next generation complex logic gate device at the molecular level^1^,^2^,^3^,^4^,^5^,^6^,^7^,^8^,^9^,^10^,^11^,^12^. Apart from its remarkably small size, this unimolecular sensor operates fast and in single mode, i.e. it requires only short incubation times and one single instrument for absorption measurements. Thus, much fewer data are produced, making the analyses very expeditious. This analytical system offers many advantages and may find an important role in occupational toxic metal hazard monitoring, molecular computing, and targeted photodynamic therapy.

## Methods

### Absorption Measurements

A solution of **1** (20 mM, 2 μL) in methanol was added to a solution of 196 μL Dulbecco’s PBS buffer/methanol (1/9) (pH = 7.3) using 96-wall plates. To this solution containing 200 μM/198 μL of **1**, a solution of a metal ion (200 mM, 2 μL) in water or urine sample was added. The mixture was allowed to equilibrate for 4–5 min. Absorbance measurements were taken at steps of 5 nm. Each absorption spectrum represented is the average of five consecutive measurements.

### Data Analysis

The absorbance experiments were repeated for all the metal ions several times. PCA was applied to distinguish between patterns generated by the absorbance intensities at different selected wavelengths in which maximal changes in intensities were observed. The PCA was able to discriminate all metal ions, different concentration of metal ions and combinations of metal ions in various samples.

## Additional Information

**How to cite this article**: Rout, B. A Miniaturized Therapeutic Chromophore for Multiple Metal Pollutant Sensing, Pathological Metal Diagnosis and Logical Computing. *Sci. Rep.* **6**, 27115; doi: 10.1038/srep27115 (2016).

## Supplementary Material

Supplementary Information
