# Supplementary material for: A Miniaturized Therapeutic Chromophore for Multiple Metal Pollutant Sensing, Pathological Metal Diagnosis and Logical Computing
Source: Sci Rep. 2016 Jun 7;6:27115. doi: 10.1038/srep27115 (PMC4895214; doi:10.1038/srep27115)
Supplement: Supplementary Information [file srep27115-s1.pdf]

A Miniaturized Therapeutic Chromophore for Multiple Metal Pollutant Sensing, Pathological Metal Diagnosis and Logical Computing

Bhimsen Rout[*,†]

[†]Organic Chemistry Division, Institute of Chemical and Engineering Sciences, A*STAR, 138665-Singapore

Contents

1. Material and methods……………………………..…………………………………….….S2

2. Effect of symmetry on Q-band of porphyrin and chlorin..………..…………………………...S2

3. Absorbance measurements ……………………………………...…….…………………….S3

3a. Solvent optimizations….…………………………………………………….………..……..S3

3b. B-band absorption response of sensor to different metal ions…………………….………......…..S4

3c. Metal identifications using Q-band absorption...……..S5

3d. Analysis of human urine samples……………………….……………………………...S5

3e. 2-Digit Keypad Lock…………………………………………………………………………S6

4. Principal Component Analysis (PCA)...……………….…………………………………..…S7

1. Material and methods

Spectroscopic grade solvents were purchased from Sigma Aldrich. Temoporfin was purchased from Xiamen Hisunny Co. Ltd. Metal chlorides such as anhydrous $ZnCl_2$ (Alfa Aesar), $FeCl_3.6H_2O$ (Sigma Aldrich), Cu(II) chloride (Sigma Aldrich), $NiCl_2.6H_2O$ (Kanto Chemicals), $CrCl_3.6H_2O$ (Sigma Aldrich), $CoCl_2.6H_2O$ (Sigma), $MnCl_{2.}4H_2O$ (Kanto Chemicals), Cd(II) chloride (Sigma Aldrich), $CaCl_2$ (Merck), Hg(II) acetate (Alfa Aesar), KCl (Sigma Aldrich), $CuSO_4.5H_2O$ (Strem Chemicals) were all of highest analytical grade. Dulbecco's PBS buffer, pH=7.3, without calcium chloride and magnesium chloride used for metal ion detection was purchased from Sigma, Singapore. Millipore water was used for spectroscopy. Human urine was collected from a healthy person. Absorbance measurements were performed on a Perkin Elmer Envision 2104 Multilabel Reader. 96-well plates were used for absorbance measurements. Principal component analysis of the absorption spectra was performed using XLSTAT version 2015.2.02.17946.

2. Effect symmetry on Q-band absorption of porphyrin and chlorin

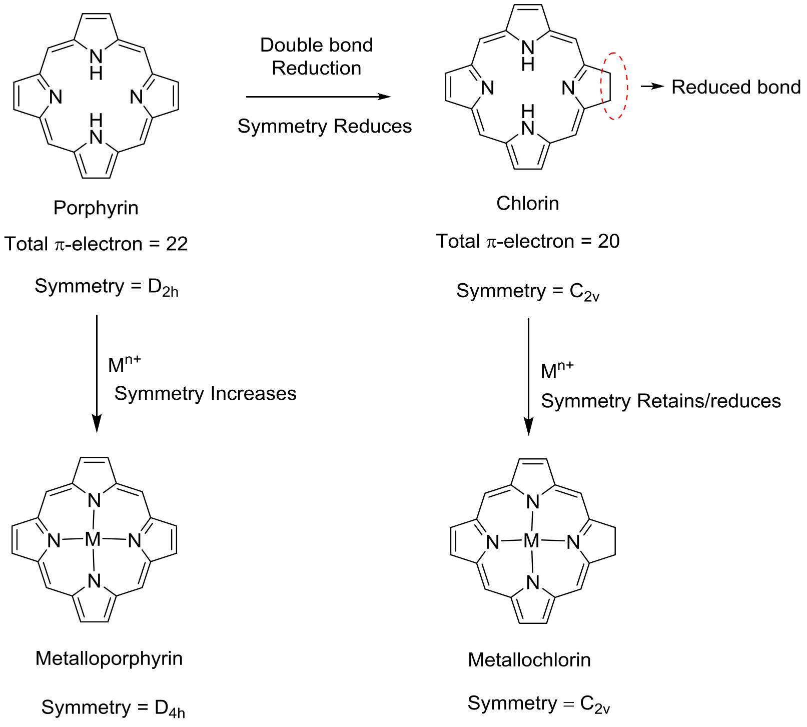

Figure S1. Effect of metallation on symmetry for porphyrin and chlorin.

Upon metallation, porphyrin loses two protons and increases symmetry from D_{2h} to D_{4h} (Fig. S1). This increase in symmetry results in less molecular vibration along X- and Y-axis of the metalloporphyrin. Hence, the intensity of Q-band absorption was reduced and only two peaks were observed. In case of chlorin which is an unsymmetrical, reduced form of porphyrin, the symmetry is reduced to C_{2v}. The unsymmetrical molecular vibration along the X- and Y-axis was increased in comparison to porphyrin, resulting in four Q-band absorption peaks. The symmetry of chlorin was further reduced by introducing a phenyl ring at the meso-position due to steric effect. Figure S2 illustrates porphyrin upon complexation with metals having different sizes, charges, binding constants resulted distortion at four pyrrole moieties of the porphyrin rings in different ways such as dome, saddle, ruffle, wave etc. which brings different molecular vibration along X- and Y-axis of the molecule and hence, generate different Q-band absorption spectra. Similar to porphyrin (Fig. S2), depending on charge and size of metal ions, several out-of-plane (Fig. 2, main text) and in-plane structural deformations can be observed. This further reduces symmetry and increases molecular vibration along the X- and Y-axis, and thus results in more Q-band peaks.

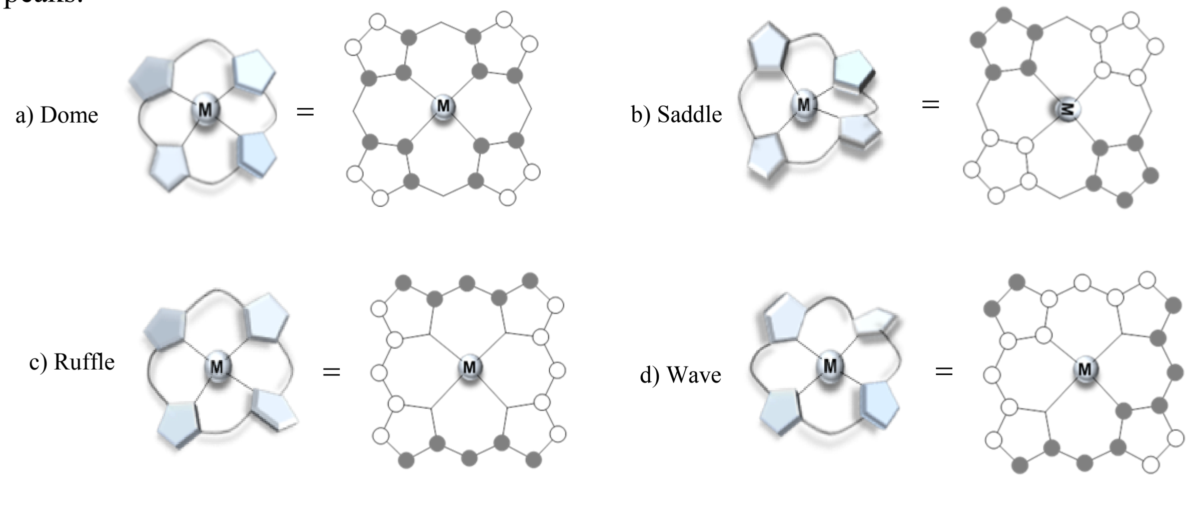

Figure S2. Schematic representation of frequently observed out-of-plane conformations of metalloporphyrins. a) Dome, b) Ruffle, c) Saddle and d) wave. Grey dots are above the plane of the molecule.

3. Absorbance measurements

3.a Solvent optimization: A solution of **1** (20 mM, 2 µL) in methanol was added to a solution of buffer (198 µL). Absorbance measurements were taken in 96-wall plates in the spectral range from 270-800 nm. The spectra were recorded at steps of 5 nm. The partially insoluble nature of sensor was observed in

both HEPES and PBS Buffer. The solvents used were HEPES buffer (pH=7.4), HEPES buffer/MeOH (v:v=1:1) (pH=7.4), HEPES buffer/MeOH (v:v=1:9) (pH=7.4), PBS buffer (pH=7.3), PBS buffer/MeOH (v:v=1:1) (pH=7.4), and PBS buffer/MeOH (v:v=1:9) (pH=7.3). The absorption intensities were analyzed at four different wavelengths: 515 nm, 540 nm, 600 nm, and 650 nm (Fig. S3). The absorption intensity was highest with PBS buffer/MeOH (v/v=1/9) (pH=7.3) (Condition F, Fig. S3) and was chosen for further metal detection. To avoid interference of metal ions present in buffer solution, Dulbecco's PBS buffer (pH=7.3), which does not contain Ca^{2+} and Mg^{2+} ions, was used for absorbance measurement in water, urine and also for keypad lock.

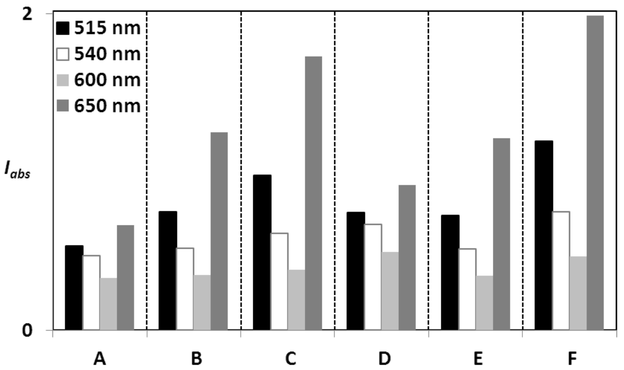

Figure S3. Absorption response of molecule **1** in: A) HEPES buffer (pH=7.4); B) HEPES buffer/methanol (1:1) (pH=7.4); C) HEPES buffer/methanol (1:9) (pH=7.4); D) PBS buffer (pH=7.3); E) PBS buffer/methanol (1:1) (pH=7.3); and F) PBS buffer/methanol (1:9) (pH=7.3) at four different wavelengths.

3.b B-band absorbance response of sensor to different metal ions: A solution of **1** (10 mM, 2 µL) in methanol was added to a solution of 196 µL PBS buffer /methanol (v/v=1/9) (pH= 7.3). To this solution containing 100 µM /198 µL of **1**, a solution of a metal ion (100 mM, 2 µL) in water was added. The mixture was allowed to equilibrate for 5 min. Absorbance measurements were taken in 96-wall plates from 370-470 nm wavelengths for B-band spectra (Fig. S4). The spectra were recorded at steps of 5 nm. The emission of the pure sensor (without metal ions) corresponds to an addition of water only.

All metal ions produced an identical B-band absorption spectrum at 410 nm, except for Cu^{2+} and Fe^{3+}. These latter metal ions exhibited a significant shift of the absorption peaks with an additional small shoulder-like peak. Hence, the B-band alone was not able to differentiate between many of the metal ions studied.

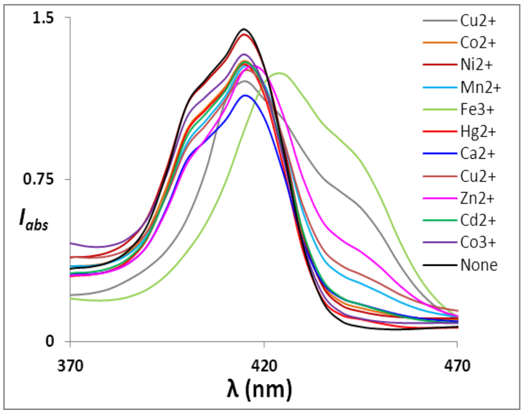

Figure S4. Absorbance spectra of **1** (100 μM) upon addition of different metal ions (1 mM) in PBS buffer/methanol (v/v=1/9) (pH= 7.3) between 370-470 nm (B-band absorption).

3.c Metal identification using Q-band: A solution of **1** (20 mM, 2 μL) in methanol was added to a solution of 196 μL Dulbecco's PBS buffer/ methanol (1/9) (pH= 7.3) using 96-wall plates. To this solution containing 200 μM /198 μL of **1**, a solution of a metal ion (200 mM, 2 μL) in water was added. The mixture was allowed to equilibrate for 4 min. Absorbance measurements were taken at steps of 5 nm. Each absorption spectrum represented is the average of five consecutive measurements (Fig. 3a, main text and Fig. S5). The emission of the pure sensor (without metal ions) corresponds to an addition of water only.

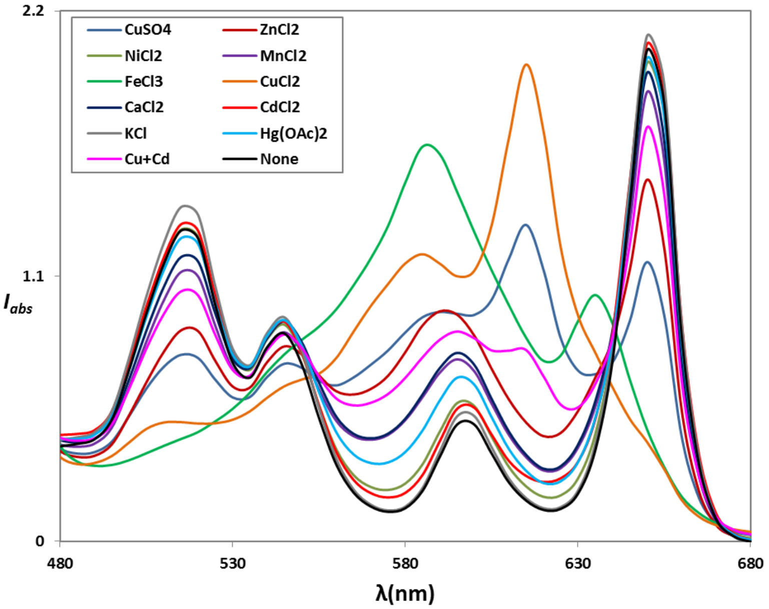

Figure S5. Q-band absorption spectra of **1** (200 μM) upon addition of different metal ions (2 mM) in Dulbecco's PBS buffer/methanol (1/9) (pH=7.3). Metals are $CuSO_4$, $CuCl_2$, $NiCl_2$, $ZnCl_2$, $MnCl_2$, $FeCl_3$, $CaCl_2$, $CdCl_2$, $Hg(OAc)_2$, KCl.

3.d Analysis of human urine samples: A solution of **1** (20 mM, 2 µL) in methanol was added to a solution of 196 µL Dulbecco's PBS buffer pH= 7.3: methanol (V:V=1:9) in 96-well plates. A solution of metal mixtures (2 µL) in human urine was added to above solution of **1** (200 µM, 198 µL). The mixture was allowed to equilibrate for 3 min. Absorbance measurements were taken at the wavelengths ranging from 270-800 nm. The spectra were recorded at steps of 5 nm as shown in figure S6. The emission of the pure sensor (without metal ions) corresponds to the addition of urine only. Each absorption spectrum represented is the average of six consecutive measurements (Fig. S6). The change in absorption intensities (ΔI_{abs} = 1- I_{abs}) from a threshold intensity ($I_{threshold}$ = 1) at four different wavelengths (e.g. 515 nm, 545 nm, 595 nm, and 650 nm) is shown in Figure 5a (main text).

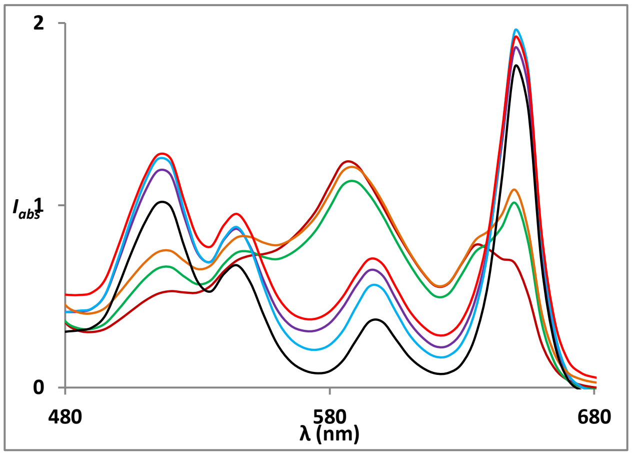

Figure S6. Change in Q-band absorption intensities generated by **1** in Dulbecco's PBS buffer / methanol (1/9) (pH 7.3) upon addition of urine containing: A) chromium (0.53 mg/mL, dark red); B) chromium (0.26 mg/mL, green); C) cobalt (0.48 mg/mL, purple); D) cobalt (0.24 mg/mL, blue); E) mixture of chromium (0.53 mg/mL) and cobalt (0.48 mg/mL, orange); F) mixture of chromium (0.26 mg/mL) and cobalt (0.24 mg/mL, red); black spectrum represents addition of urine only.

3.e 2-Digit Keypad Lock: A solution of **1** (20 mM, 2 µL) in methanol was added to a solution of 196 µL Dulbecco's PBS buffer/ methanol (1/9) (pH= 7.3) in 96-wall plates. A solution of a metal M0 (200 mM, 2 µL) in water as first input key was added to the above solution of **1** (200 µM, 198 µL). The mixture was allowed to equilibrate for 3 min. Subsequently, a solution of another metal M1 (200 mM, 2uL) in water was added as second input key and equilibrated for another 3 min. Absorbance measurement were taken at the wavelengths ranging from 270-800 nm. The spectra were recorded at steps of 5 nm. Each absorption spectrum represented is the average of five consecutive measurements (Fig. 7a, main

text). The absorption of the pure sensor (without metal ions) corresponds to an addition of water only. The two metals used in this case were $FeCl_3$ (M0) and $CuCl_2$ (M1). The highly secure 1-digit and 2-digit passwords are M0, M1, M0M0, M1M1, M0M1, and M1M0.

Few other metal combinations such Zn-Fe, Fe-Zn, Zn-Cu and Cu-Zn were tested in different sequence (Fig. S7). Zinc and iron metal in different sequence were producing same absorption signature whereas zinc and copper producing different absorption spectrum. It was observed that resultant absorption spectrum also depends on metal types along with concentration of metal and incubation time.

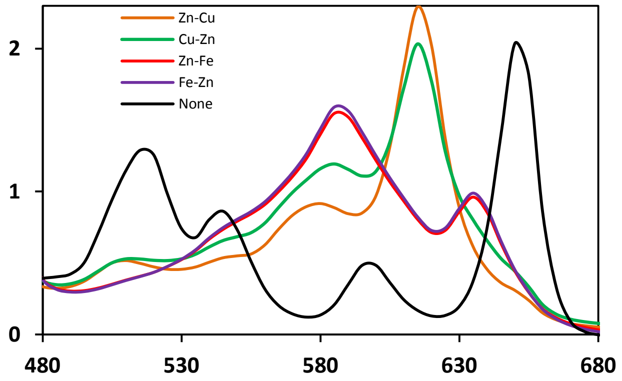

Figure S7. Change in Q-band absorption intensities generated by **1** in Dulbecco's PBS buffer / methanol (1/9) (pH 7.3) upon addition of zinc (2 mM) and Fe (2mM) or Cu (2mM) in different sequence.

4. Principal Component Analysis (PCA).

The absorbance experiments were repeated for all the metal ions as Figure S3. A combination of 200 mM cobalt chloride and 200 mM cadmium chloride, or a combination of 200 mM iron chloride and 100 mM nickel chloride were used to study discrimination for mixture of metals. Both 200 mM or 100 mM solution of zinc chloride or iron chloride were used to study discrimination between different concentrations of metals. PCA was applied to distinguish between patterns generated by the absorbance intensities at six different wavelengths (e.g. 515 nm, 545 nm, 575 nm, 600 nm, 625 nm and 650 nm) in which maximal changes in intensities were observed. The PCA was able to discriminate all metal ions, different concentration of metal ions and combinations of toxic metal ions (Fig. 4, main text). The training set PCA was done taking these six wavelengths and color change further assisted to increase the accuracy (Table S1). Similarly, PCA differentiation between human urine containing cobalt and chromium metal

ions at different concentration and combinations (Figure 5b, main text) was achieved by analyzing change in absorption intensities (ΔI_{abs} = 1- I_{abs}) at four different wavelengths (e.g. 515 nm, 545 nm, 595 nm, and 650 nm). The training set PCA was done at these four wavelengths (Table S2). Finally, PCA differentiation between six different 1-digit and 2-digit chemical passwords M0, M1, M0M0, M1M1, M0M1, and M1M0 were analyzed at six different wavelengths such as 510 nm, 550 nm, 575 nm, 590 nm, 615 nm and 640 nm (Fig. 7b, main text). Principal component analysis of the absorption spectra was performed using XLSTAT version 2015.2.02.17946.

Table S1: Error analysis training set for metal analysis:

Input analytes	Factor 1	Factor 2	Detected metals
$CuSO_4$	-1.34681	-0.30972	$CuSO_4$
$CuSO_4$	-1.31172	-0.20003	$CuSO_4$
$CuSO_4$	-1.59907	-0.69495	$CuSO_4$
$CuSO_4$	-1.50477	-0.4814	$CuSO_4$
$CuSO_4$	-1.70248	-0.15055	$CuSO_4$
$CuSO_4$	-1.49297	-0.36733	$CuSO_4$
Zn	0.021281	0.252846	Zn
Zn	-0.02571	0.178124	Zn
Zn	-0.02558	-0.00249	Zn
Zn	-0.13166	-0.1829	Zn
Zn	-0.06749	-0.10945	Zn
Ni	2.566356	-0.13965	Ni
Ni	2.557895	-0.12653	Ni
Ni	2.554885	-0.20714	Ni
Ni	2.861859	0.504199	**Hg/K**
Ni	2.512047	-0.25603	Ni

Mn	1.491246	0.129412	Mn
Mn	1.471376	0.031845	Mn
Mn	1.518427	0.138367	Mn
Mn	1.553351	0.175899	Mn
Mn	1.432998	-0.06271	Mn
Fe (2mM)	-3.32128	1.028416	Fe (2mM)
Fe (2mM)	-3.35133	1.027623	Fe (2mM)
Fe (2mM)	-3.32239	0.512999	**Fe (1mM)**
Fe (2mM)	-3.32614	1.285376	Fe (2mM)
Fe (2mM)	-3.31384	0.874267	Fe (2mM)
$CuCl_2$	-3.64634	-0.87762	$CuCl_2$
$CuCl_2$	-3.91318	-0.37818	$CuCl_2$
$CuCl_2$	-3.90998	0.267924	**$CuCl_2$/Fe**
$CuCl_2$	-4.02883	-0.17211	$CuCl_2$
$CuCl_2$	-3.83408	-0.57524	$CuCl_2$
Ca	1.588741	0.288435	**Mn**
Ca	1.484033	0.055379	**Mn**
Ca	1.782219	0.672587	Ca
Ca	1.727425	0.420948	Ca
Ca	1.816402	0.589313	Ca
Ca	1.679764	0.405332	Ca
Cd	1.622917	-1.22565	Cd
Cd	1.84013	-0.7212	Cd
Cd	1.743204	-0.94696	Cd
Cd	1.827798	-0.79963	Cd

Cd	1.923751	-0.65027	Cd
Cd	1.637894	-1.31327	Cd
Cd	1.765949	-0.94283	Cd
Hg	2.214711	0.303548	Hg
Hg	2.392707	0.711334	Hg
Hg	2.317847	0.431671	Hg
Hg	2.148663	0.100487	Hg
Hg	2.32784	0.468312	Hg
Hg	2.197831	0.192571	Hg
K	3.246758	0.282783	K
K	3.227746	0.224297	K
K	3.126642	-0.01625	K
K	3.132469	0.006859	K
K	3.164521	0.088547	K
K	3.056142	-0.13172	K
Cu+Cd	0.180238	-0.27641	**Zn**
Cu+Cd	0.773193	0.988969	Cu+Cd
Cu+Cd	0.3231	0.304515	Cu+Cd
Cu+Cd	0.206538	0.715624	Cu+Cd
Cu+Cd	0.581607	0.807172	Cu+Cd
Cu+Cd	0.425351	0.341885	Cu+Cd
Zn (1mM)	1.093644	-0.49587	Zn (1mM)
Zn (1mM)	0.992966	-0.09357	Zn (1mM)
Zn (1mM)	1.367514	0.858028	**Ca**
Zn (1mM)	1.073042	0.004233	Zn (1mM)

Zn (1mM)	1.131791	0.068206	Zn (1mM)
Fe (1mM)	-3.1666	0.3393	Fe (1mM)
Fe (1mM)	-3.15627	0.381266	Fe (1mM)
Fe (1mM)	-3.1572	0.729635	Fe (1mM)
Fe (1mM)	-3.16699	0.574527	Fe (1mM)
Ni+Fe	-3.16177	1.724359	**Fe (2mM)**
Ni+Fe	-3.13772	-0.27102	Ni+Fe
Ni+Fe	-3.05431	-0.42548	Ni+Fe
Ni+Fe	-3.03441	-0.77587	Ni+Fe
Cr	-2.22219	-1.10063	Cr
Cr	-2.26138	-0.5977	Cr
Cr	-2.23074	-0.57923	Cr
Cr	-2.24817	-1.278	Cr
Cr	-2.27478	-0.88398	Cr
Cr	-2.23661	-0.66786	Cr

Table S2: Error Analysis of metal concentrations and combinations in Urine

Input Analytes	F1	F2	Detected metals
Cr 2mM	2.720321	0.428563	Cr 2mM
Cr 2mM	2.640637	-0.03951	Cr 2mM
Cr 2mM	2.604719	-0.05144	Cr 2mM
Cr 2mM	2.794649	0.580295	Cr 2mM
Cr 2mM	2.729827	0.212121	Cr 2mM
Cr 1mM	1.840553	0.369633	Cr 1mM
Cr 1mM	1.684451	-0.12972	Cr 1mM

Cr 1mM	1.799182	-0.24228	Cr 1mM
Cr 1mM	2.096242	0.566643	Cr 1mM
Cr 1mM	1.861253	0.087531	Cr 1mM
Co 2mM	-1.39608	0.313616	Co 2mM
Co 2mM	-1.47167	0.240753	Co 2mM
Co 2mM	-1.29512	0.547399	Co 2mM
Co 2mM	-1.22382	0.668233	Co 2mM
Co 2mM	-1.42518	0.28638	Co 2mM
Co 1mM	-2.13608	0.09147	Co 1mM
Co 1mM	-2.26952	-0.0715	Co 1mM
Co 1mM	-1.88662	0.296034	Co 1mM
Co 1mM	-1.83804	0.574508	Co 1mM
Co 1mM	-1.89261	0.481218	Co 1mM
Co 1mM	-1.33404	1.28563	**Co 1mM/Co 2mM**
Cr 2mM-Co 2mM	1.104522	-1.04677	Cr 2mM-Co 2mM
Cr 2mM-Co 2mM	1.124381	-1.11587	Cr 2mM-Co 2mM
Cr 2mM-Co 2mM	1.581305	-0.54352	Cr 2mM-Co 2mM
Cr 2mM-Co 2mM	1.721266	-0.08768	**Cr 1mM**
Cr 2mM-Co 2mM	1.421832	-0.50515	Cr 2mM-Co 2mM
Cr 1mM-Co 1mM	-2.05267	-0.62751	Cr 1mM-Co 1mM
Cr 1mM-Co 1mM	-2.11841	-1.12863	Cr 1mM-Co 1mM
Cr 1mM-Co 1mM	-2.04646	-0.86242	Cr 1mM-Co 1mM
Cr 1mM-Co 1mM	-2.10556	-0.55407	Cr 1mM-Co 1mM
Cr 1mM-Co 1mM	-1.57102	-0.16816	Cr 1mM-Co 1mM
Cr 1mM-Co 1mM	-1.66225	0.144209	Cr 1mM-Co 1mM
